# Comparison between the Hypolipidemic Activity of Parsley and Carob in Hypercholesterolemic Male Rats

**DOI:** 10.1155/2017/3098745

**Published:** 2017-09-28

**Authors:** Haddad A. El Rabey, Madeha N. Al-Seeni, Habibah B. Al-Ghamdi

**Affiliations:** ^1^Biochemistry Department, Faculty of Science, King Abdulaziz University, Jeddah, Saudi Arabia; ^2^Bioinformatics Department, Genetic Engineering and Biotechnology Research Institute, University of Sadat City, P.O. Box 79, Sadat City, Egypt

## Abstract

Hypercholesterolemia is commonly associated with obesity that leads to heart diseases and diabetes. The hepatocardioprotective activity of parsley and carob methanol extract was tested in hypercholesterolemic male rats. Twenty-four male albino rats were divided into four groups (*n* = 6). Group 1 was the negative control group fed with fat rich diet, group 2 (G2) was hypercholesterolemic rats fed with fat rich diet with 2% cholesterol, and group 3 and group 4 (G3 and G4) were hypercholesterolemic rats supplemented with 2% cholesterol and cotreated with 20% w/w parsley seed methanol extract and 20% w/w carob legume methanol extract, respectively. The experiment was conducted for eight weeks. The positive hypercholesterolemic rats showed significant increase in serum levels of total cholesterol, triglycerides, low density lipoprotein (LDL), very low density lipoprotein (VLDL), lactate dehydrogenase (LDH), creatine kinase-mb, liver function enzymes, and decrease in the high density lipoproteins (HDL). Moreover, heart and liver tissues were ameliorated and nearly restored their normal appearance. It could be concluded that both parsley and carob extracts supplementations have a protective effect against hyperlipidemia and improved the histological alteration in heart and liver tissues. The methanol extract of parsley appeared to be more efficient than that of carob in lowering hypercholesterolemia.

## 1. Introduction

Hypercholesterolemia represents a major risk factor for cardiovascular disease (CVD) which continues to remain a significant problem in developed countries and is a growing health concern worldwide [1, 2]. Taking a high-fat diet and other lifestyle factors like being overweight, smoking, heavy alcohol use, and lack of exercise have been associated with some cancers, for example, postmenopausal breast cancer, prostate cancer, and bowel cancer [3].

Patients with elevated lipids should attempt to correct this abnormality via lifestyle modification, for example, adjustment in diet by minimizing consumption of saturated fat and cholesterol and increase in physical activity [4]. Consumption of soluble fiber such as oat bran and barley bran can lower LDL cholesterol levels [5–7] associated with significant reduction in risk for cardiovascular disease.

Consumption of foods that contain antioxidant compounds protects the low (LDL) and very low density lipoproteins from oxidation, reduces lipid levels in plasma, and thus may reduce risk of cardiovascular disease [8].

Parsley (*Petroselinum hortense* Mill.) seed is rich in vitamins including A and C, as well as thiamine, riboflavin, niacin, and minerals including calcium, zinc, potassium, and iron [9]. It also possesses a number of medicinal benefits including antimicrobial, antianemic, menorrhagic, anticoagulant, antihyperlipidemic, antihepatotoxic, antihypertensive, diuretic effects, hypoglycaemic, hypouricemic, antioxidative, and estrogenic activities [10] due to its content of flavonoids, coumarins, ascorbic acid, carotenoids, various terpenoic compounds, phenyl propanoids, phathalides, and tocopherol. Parsley seeds are also rich in monoterpenes (in particular *α*- and *β*-pinene) and oxygenated phenylpropenes, which constitute the greater part of the oil [11–13].

Carob legumes have a marked nutritional value due to its high dietary fiber and phenol compounds which exert a preventative role against heart disease and lowering serum cholesterol [14, 15]. It also contains minerals, amino acids, sugars, and dietary fibers including lignin and polyphenols; flavonol glycoside, 4′-p-hydroxybenzoylisorhamnetin-3-O-*α*-L-rhamnopyranoside named ceratoside, together with the known kaempferol-3-O-*α*-L-rhamnopyranoside (afzelin), quercetin-3-O-*α*-L-arabinofuranoside (auriculain), quercetin-3-O-*α* L-rhamnopyranoside, *β*-sitosterol, and *β*-sitosterol-3-O-*β*-D-glucoside were isolated from carob seeds [14, 16, 17]. Moreover, carob bean gum increases the dietary fiber in food products without increasing the calories and increases the swelling of the food once in the stomach, which encourages a feeling of fullness, so it is considered a natural appetite suppressant and effective in prevention and treatment of hypercholesterolemia [18, 19].

The current study aimed to evaluate the hypolipidemic and antioxidant activity of parsley and carob methanol extract and their potential in protecting heart and liver in hypercholesterolemic male rats.

## 2. Materials and Methods

### 2.1. Plant Materials

Parsley seeds and carob legumes were purchased from a local herbal medicine shop in Jeddah, Saudi Arabia, identified by Professor Haddad El Rabey (Botanist), and a specimen was deposited in Biochemistry Department, Faculty of Science, KAU, KSA.

### 2.2. The Lipid Rich Diet

The lipid rich diet consisted of the following: 16% casein, 10% corn oil, 4% N.N cellulose, 4% salt mixture, 1% vitamin mixture, 0.2% choline chloride, 0.2% DL-methionine, and 64.6% corn starch [20].

### 2.3. Animals and Housing Conditions

Twenty-four male albino rats* (Rattus norvegicus)* of East China Origin weighing 150–200 g were obtained from Faculty of Pharmacy, King Abdulaziz University, Jeddah, Saudi Arabia. All the animal experiments were carried out under protocols approved by the Institutional Animal House of the University of King Abdulaziz at Jeddah, Saudi Arabia. Animals were housed six per polycarbonate cage. Cages, bedding, and glass water bottles were replaced twice per week. The stainless steel feed containers were changed once a week.

### 2.4. Preparation of Methanol Extract

The methanol extract was prepared by soaking 200 g of each of the dry parsley seed powder and carob legumes powder in 1 liter of 90% methyl alcohol with shaking for 5 days and then kept in a refrigerator. The methanol was evaporated using a rotatory evaporator apparatus attached with a vacuum pump. Twenty grams of the semisolid methyl extract was suspended in 100 ml distilled water with 2 ml of tween 80 (as a suspending agent) to prepare 20% alcohol [21].

### 2.5. Experiment Design

The tested animals were fed with the lipid rich diet and kept under observation for 2 weeks before the start of the experiment to exclude any undercurrent infection. The rats were then divided randomly into four groups each of 6 rats as follows: the first group (G1) was the normal untreated control group fed with normal diet, the second group (G2) was fed with 2% cholesterol in the diet to induce hypercholesterolemia, the third group (G3) was fed with 2% cholesterol and cotreated with 20% parsley seeds methanol extract using stomach tube, and the fourth group (G4) was fed with 2% cholesterol and cotreated with 20% carob legumes methanol extract using stomach tube. The experiment was conducted for 8 weeks as an adequate period to induce hypercholesterolemia [22].

### 2.6. Physiological Evaluation

The following biological parameters were estimated: daily water consumption, daily food intake, daily body weight gain (BWG), percentage of body weight gain (BWG%), food efficiency ratio (FER), percentage of food efficiency ratio (FER%), and organs weight [23].

### 2.7. Dissection and Blood Collection

At the end of the experiment, animals were fasted 14–16 hours after their last feeding and blood was collected from the heart of dimethyl-ether preanaesthetized rats in plain tubes for biochemical analysis. Blood serum was obtained by centrifugation at 1000 rpm for 10 min at room temperature and then stored at −20°C until analysis was performed. Animals were sacrificed by cervical dislocation, and then the abdomen was dissected and the heart, the liver, the two kidneys, and two testes were rapidly excised and weighed. Parts of heart and liver were saved in ice-cold for antioxidant enzymes and lipid peroxidation estimation in liver and heart tissue homogenate. The other parts of the liver and the heart were saved in saline solution for histopathological investigations.

### 2.8. Lipid Profile

Serum total cholesterol (TC) and triglycerides (TG) were determined by colorimetric methods as described by Young [24] using Spinreact Kit (Spain) according to the instruction of the supplier. Serum high density lipoprotein (HDL) was estimated according to the colorimetric method of Naito [25] using Spinreact Kit (Spain) according to the instruction of the supplier. Serum LDL and VLDL were calculated according to the equation of Srivastava et al. [26] as follows: LDL = TC − (HDL + TG/5) and VLDL = TC − (LDL + HDL).

### 2.9. Liver Enzymes

Serum alanine transaminase (ALT) and alkaline phosphatase (ALP) were estimated spectrophotometrically according to the method of Thefeld et al. [27] and Schlebusch et al. [28], respectively, using Human Kit (Germany) according to the instruction of the supplier. Serum aspartate aminotransferase (AST) was estimated spectrophotometrically according to the method of Thefeld et al. [27] using Swemed Diagnostics kit (India).

### 2.10. Lactate Dehydrogenase

The activity of lactate dehydrogenase (LDH) enzyme was estimated spectrophotometrically using Teco Diagnostics Kit (USA) as described by Martinek [29] according to the instruction of the supplier.

### 2.11. Creatine Kinase-MB

Serum creatine kinase-MB (CK-MB) was estimated using the Enzyme Immunoassay method as described by Lee and Goldman [30] using Oxis kit from Oxis International Inc. (USA) according to the instruction of the supplier.

### 2.12. Histopathological Investigations

Parts of liver and heart were washed in sterile saline and fixed in 10% neutral formalin for histopathological studies. Tissues were then dehydrated in gradual ethanol (50–99%), cleared in xylene, and embedded in paraffin. Sections were prepared and then stained with hematoxylin and eosin (H&S) dye for microscopic investigation [31]. The stained sections were examined and photographed under an Olympus light microscope equipped with a digital camera.

### 2.13. Statistical Analysis

Mean values, standard error, and test of significance were calculated using SPSS program (SPSS version 17, Oxford University, 2009-2010, UK). The least significant value and analysis of variance were calculated using SAS package.

## 3. Results

### 3.1. Organ Weight

Table 1 shows the effect of administration of 20% (w/w) parsley and carob methanol extracts for 8 weeks on the weight of heart, liver, right kidney, and left kidney in hypercholesterolemic rats for two months. The mean values of the liver and the heart weights were decreased as a result of feeding rats in G2 with 2% cholesterol in diet for 8 weeks. While the mean values of the right and the left kidney were increased as a result of feeding rats in G2 with 2% cholesterol in diet for 8 weeks, compared with the negative control. After treatment with 20% parsley seeds methanol extract in G3, the mean values of the heart, left, and right kidney weight were nonsignificantly decreased, while the mean value of the liver weight was nonsignificantly increased compared with the positive control group. In G4 which received 20% carob legume methanol extract with diet, the mean value of the heart weight was nonsignificantly increased while the mean value of the liver weight was significantly decreased compared with the positive control group. The mean value of the kidney weight in G4 did not show significant changes.

### 3.2. Physiological Parameters

Table 2 shows the effect of parsley and carob methanol extracts administration for 8 weeks on physiological evaluations in hypercholesterolemic male rats. No significant difference was recoded due to hypercholesterolemia or treating the hypercholesterolemic rats with parsley or carob methanol extracts.

### 3.3. Lipid Profile

Table 3 shows the effect of administration of 20% (w/w) parsley and carob methanol extracts for 8 weeks on serum lipids in hypercholesterolemic male rats. The mean values of the serum total cholesterol, serum triglycerides, serum low density lipoproteins, and serum very low density lipoproteins significantly increase (at 0.001) in group 2 who received 2% cholesterol for 8 weeks. While the mean value of serum high density lipoproteins significantly decreases compared with the negative control group. The concurrent supplementation with 20% parsley seeds methanol extract in G3 and 20% carob legume methanol extract in G4 to the hypercholesterolemic rats significantly ameliorated all lipids parameters by decreasing serum TC, TG, LDL, and VLDL and increasing serum HDL.

### 3.4. Liver Enzymes

Table 4 shows the effect of administration of 20% (w/w) parsley and carob methanol extracts for 8 weeks on serum liver enzymes in hypercholesterolemic rats for 8 weeks.

The mean values of ALT, AST, and ALP were significantly (at *P* < 0.001) increased in group 2 as a result of cholesterol supplementation for 8 weeks. The concurrent supplementation with 20% parsley seeds methanol extract in G3 and 20% carob legume methanol extract in G4 to the hypercholesterolemic rats significantly (at *P* < 0.001) decreased all liver enzymes during experiment period. Parsley seeds methanol extract in G3 ameliorated the liver enzymes more than carob legume methanol extract in G4.

### 3.5. Lactate Dehydrogenase

Table 4 also shows the effect of administration of 20% (w/w) parsley and carob methanol extracts for 8 months on serum lactate dehydrogenase in hypercholesterolemic rats for two months. The mean value of serum lactate dehydrogenase activity was significantly (at *P* < 0.001) increased in the positive control group (G2), as a result of cholesterol administration for 8 weeks. The concurrent treatment with 20% parsley seeds methanol extract in G3 and 20% carob legume methanol extract in G4 for 8 weeks significantly (at *P* < 0.001) decreased lactate dehydrogenase levels in the serum of hypercholesterolemic rats under study. Parsley seeds methanol extract in G3 decreased lactate dehydrogenase more than carob legume methanol extract.

### 3.6. Creatine Kinase-MB

Table 4 also shows the effect of administration of 20% (w/w) of parsley and carob methanol extract for 8 weeks on serum creatine kinase-MB in hypercholesterolemic male rats. The serum creatine kinase-MB level was nonsignificantly increased as a result of induced hypercholesterolemia in G2. The concurrent treatment with 20% parsley seeds methanol extract in G3 and 20% carob legume methanol extract in G4 for 8 weeks decreased the creatine kinase-MB and restored it to its normal levels in G1.

### 3.7. Histopathological Investigations

#### 3.7.1. Histopathology of the Liver

**Figure 1:**
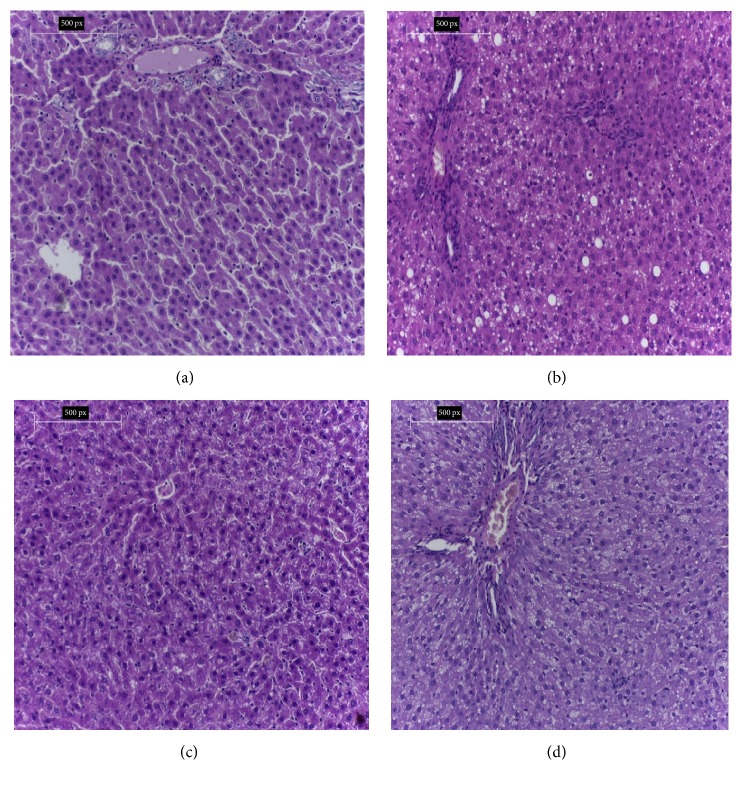
(a) Hepatic tissues of rat from the negative control group showing normal hepatic tissue. (b) Hepatic tissue of rat from hypercholesterolemic positive control group showing ballooning of hepatocytes, fatty changes, and inflammation. (c) Hepatic tissues photomicrography of hypercholesterolemic rat from group (3) pretreated parsley methanol extract, nearly showing normal hepatocytes. (d) Hepatic tissues of hypercholesterolemic rat from group (4) treated with carob methanol extract, showing nearly normal hepatocytes (H&E. ×200).

Figure 1 shows histology of liver of rats under study.  Figure 1(a) shows the hepatic tissue of the negative control group with normal architecture and normal hepatocytes. The portal tract shows normal bile duct, portal vein, and hepatic artery.  Figure 1(b) shows the hepatic tissue of the hypercholesterolemic rats in the positive control group fed with 2% cholesterol for 8 weeks with drastic alteration in architecture such as liver ballooning of hepatocytes and fatty changes, which made the hepatocytes disrupted as a result of lipid accumulation. Inflammatory cellular infiltration was also detected around the blood vessels. After treatment with parsley methanol extract in group (3) for 8 weeks, the liver showed moderate recovery from cellular damage, with nearly normal hepatocytes as shown in Figure 1(c). The liver sections of carob methanol extract treated group (G4) seemed to be restoring the normal appearance and showed minor effect of fatty changes and mild inflammation with normal hepatocytes as shown in Figure 1(d).

#### 3.7.2. Histopathology of the Heart

**Figure 2:**
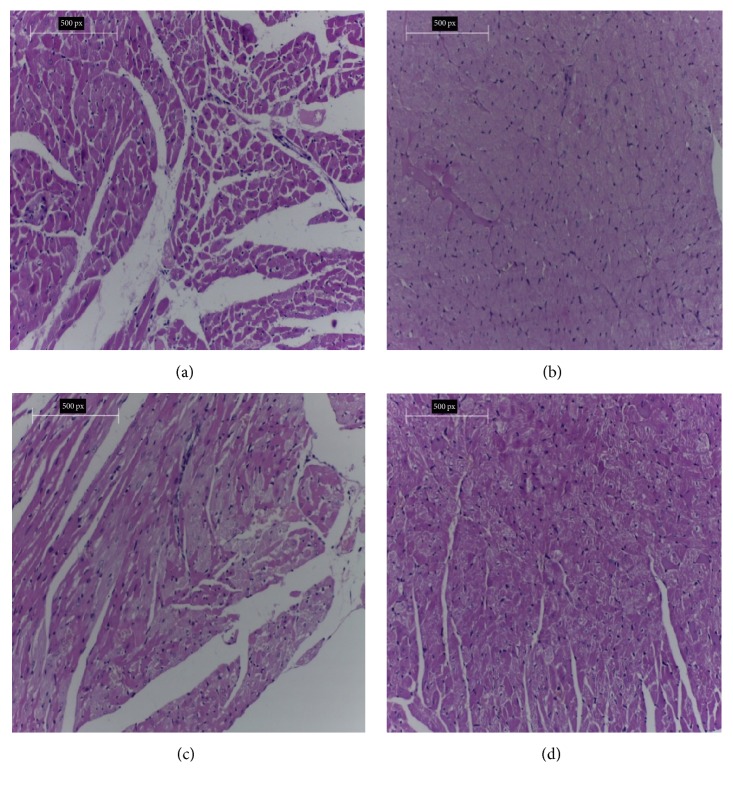
(a) Cardiac tissues of rats from the negative control group showing normal cardiac tissues. (b) Cardiac tissues of hypercholesterolemic rat from the positive control group showing congestion and marked degeneration of myocardial muscles with ballooning and degeneration. (c) Cardiac tissues of hypercholesterolemic rat from group (3) treated with parsley methanol extract, nearly restored their normal cardiac structure. (d) Cardiac tissues of hypercholesterolemic rat from group (4) treated with carob methanol extract, nearly restored their normal structure (H&E. ×200).

Figure 2 shows histology of heart of rats under study.  Figure 2(a) shows cardiac tissue of the negative control group with normal architecture of cardiac tissue and myocardial muscles. No histopathological changes were noticed.  Figure 2(b) shows cardiac tissue of hypercholesterolemic rats of the positive control group fed with 2% cholesterol for 8 weeks showing changes in cardiac structure showing congestion and marked degeneration of myocardial muscles with ballooning and degeneration of cardiocytes. After treatment with parsley methanol extract in G3 for 8 weeks, the cardiac tissues showed nearly normal tissues with regenerating myocardial cells as shown in Figure 2(c). Similarly, the cardiac tissues of rats in G4 which were treated with carob methanol extract showed nearly normal tissues with residual degeneration with areas of regenerating of myocardial cells as shown in Figure 2(d).

## 4. Discussion

The present study was focused on studying the antioxidant and hypolipidemic activity of 20% (w/w) parsley seeds and carob legumes methanol extract supplementation for 8 weeks in hypercholesterolemic male rats for a probable hepatocardioprotection. The hypercholesterolemic male rats (G2) treated with cholesterol (2% w/w for 8 weeks) showed a significant increase in lipid profile (total cholesterol, triglyceride, low density lipid-cholesterol, and very low density lipid-cholesterol) and a significant decrease in high density lipid-cholesterol. This result is consistent with previous study [6, 7, 32]. The concurrent oral administration of 20% parsley seeds methanol extract in G3 and 20% carob legume methanol extract in G4 to hypercholesterolemic rats for 8 weeks significantly improved the serum lipid profile parameters by decreasing the total cholesterol, TG, LDLc, and VLDLc and increasing HDLc. This result agrees with that of Ruiz-Roso et al. [33]. The hypolipidemic activity of parsley may be due to the presence of glucosinolates, betalains, plant proteins, carotenoids, and phenolic compounds [34]. It is also rich in antioxidant compounds including flavonoids, carotenoids, and other phenolic compounds [9, 35]. On the other hand, carob is also rich in phenolic antioxidants and minerals, in addition to dietary fibers [14, 16, 36].

Liver function parameters (serum aspartate aminotransferase, serum alanine aminotransferase, and serum alkaline phosphatase) were significantly increased in the positive control group due to induced hypercholesterolemia, compared with the negative control. This result is consistent with that of El Rabey et al. [6] and Abulnaja and El Rabey [7]. The concurrent treatment with 20% parsley seeds methanol extract in G3 and 20% carob legume methanol extract in G4 for 8 weeks significantly improved the liver functions by decreasing the studied liver enzymes activity. Parsley seeds methanol extract showed better effect than that of carob legume. In contrast, there were no other significant differences in AST, ALP, and ALT between the beginning and the end of treatment in the subjects of insoluble dietary fiber from carob pulp preparation [33].

The markers of heart damage; lactate dehydrogenase (LDH); and creatine kinase-MB were increased under induced hyperlipidemia condition in G2 rats. Abulnaja and El Rabey [7] stated that lactate dehydrogenase is released during heart tissue damage resulting from hypercholesterolemia. The decrease of LDH as a result of the concurrent treatment with 20% parsley seeds methanol extract in G3 for 8 agrees with previous investigations [37]. Creatine kinase-MB (CK-MB) enzyme was slightly affected during the experiment but its level remained within the normal range even after treatment in G3 and G4.

The hepatic section of hypercholesterolemic rats showed disarrangement and inflammation as a result of hypercholesterolemia. Also, the heart section of hypercholesterolemic rats showed marked degeneration of myocardial muscles with ballooning of cardiomyocytes. This result is consistent with other studies showing that there is a relationship between hypercholesterolemia and pathological alteration of vital organs [7, 38]. The concurrent treatment with 20% parsley seeds methanol extract in G3 hypercholesterolemic rats and 20% carob legume methanol extract in G4 hypercholesterolemic rats for 8 weeks significantly improved the liver tissues and the heart tissue and nearly restored them to their normal state. This result is consistent with that of Kamal et al. [38].

Both parsley and carob have a protective role against many of the pathological changes due to their higher content of antioxidant substance such as flavonoids and phenolic compounds [36, 39]. Other natural products contained in olive oil and* Nigella sativa* also shoed hepatoprotective effect against tetrachlorocarbon hepatotoxicity [40].

It could be concluded that both parsley and carob succeeded in lowering the lipid profile levels and oxidative stress in hypercholesterolemic rats, but parsley appeared to be more efficient than carob. Moreover, the current study suggested that both parsley and carob improved the adverse conditions in hypercholesterolemic rats as shown in the heart and liver histological results. In future investigation, HPLC analysis of both extracts should be achieved in order to determine the active constituents that lowered blood lipids and protected both heart and liver.

## Figures and Tables

**Table 1:** Effect of parsley and carob methanol extracts administration for 8 weeks on organ weight in hypercholesterolemic male rats.

Organs weight g	Statistics	G1 negative control	G2 positive control	G3 20% w/w parsley methanol extract	G4 20% w/w carob methanol extract
Heart	Mean ± SE	0.966 ± 0.033^a^	0.833 ± 0.061^a^	0.800 ± 0.044^a^	0.933 ± 0.095^a^
	LSD 0.05 = 0.212				
	*T*-test	—	1.451^NS^	0.363^NS^	−0.732^NS^

Liver	Mean ± SE	10.400 ± 0.687^a^	9.050 ± 1.720^a^	10.733 ± 0.462^a^	7.250 ± 1.279^b^
	LSD 0.05 = 2.551				
	*T*-test	—	0.762^NS^	−0.882^NS^	0.916^NS^

Right kidney	Mean ± SE	0.583 ± 0.030^a^	0.633 ± 0.0210^a^	0.666 ± 0.042^a^	0.633 ± 0.021^a^
	LSD 0.05 = 0.090				
	*T*-test	—	−2.236^NS^	−0.598^NS^	0.000^NS^

Left kidney	Mean ± SE	0.616 ± 0.0166^a^	0.683 ± 0.016^a^	0.633 ± 0.033^a^	0.633 ± 0.021^a^
	LSD 0.05 = 0.077				
	*T*-test	—	−3.162^*∗∗*^	1.168^NS^	1.464^NS^

Right testis	Mean ± SE	1.216 ± 0.110^a^	0.965 ± 0.020^b^	1.183 ± 0.030^a^	1.116 ± 0.030^ab^
	LSD 0.05 = 0.190				
	*T*-test	—	2.272^*∗*^	−5.454^*∗∗∗*^	−4.373^*∗∗∗*^

Left testis	Mean ± SE	1.266 ± 0.120^a^	0.956 ± 0.019^b^	1.1500 ± 0.050^ab^	1.200 ± 0.025^a^
	LSD 0.05 = 0.209				
	*T*-test	—	2.671^*∗∗*^	−3.15^*∗∗*^	−5.883^*∗∗∗*^

Data are represented as mean ± SE. *T*-test values ^*∗*^significant at *P* < 0.05, ^*∗∗*^significant at *P* < 0.01, and ^*∗∗∗*^significant at *P* < 0.001. ANOVA analysis: within each row, means with different superscript (a, b, c, or d) are significantly different at *P* < 0.05, whereas means superscripts with the same letters mean that there is no significant difference at *P* < 0.05. LSD: least significant difference; NS: nonsignificant.

**Table 2:** Effect of administration of 20% (w/w) parsley and carob methanol extracts for 8 weeks on food intake (FI) body weight gain (BWG) and food efficiency ratio (FER) in hypercholesterolemic male rats.

Physiological evaluation	Statistics	G1 negative control	G2 positive control	G3 20% w/w parsley methanol extract	G4 20% w/w carob methanol extract
BWG g/8 weeks	Mean ± SE	33.16 ± 1.641^a^	29.16 ± 0.872^a^	31.33 ± 0.843^a^	29.00 ± 1.238^a^
	LSD 0.05 = 3.781				
	*T*-test	^**—**^	1.743^NS^	−2.381^NS^	0.150^NS^

BWG g/day	Mean ± SE	0.551 ± 0.027^a^	0.484 ± 0.014^b^	0.520 ± 0.015^NS^	0.482 ± 0.020^a^
	LSD 0.05 = 0.063				
	*T*-test	^**—**^	1.729^NS^	−2.270^NS^	0.134^NS^

BWG %	Mean ± SE	17.85 ± 0.872^a^	16.08 ± 0.479^a^	16.39 ± 0.444^a^	14.74 ± 0.683^a^
	LSD 0.05 = 2.045				
	*T*-test	^**—**^	1.423^NS^	−0.670^NS^	2.362^NS^

FER g/day	Mean ± SE	0.031 ± 0.001^a^	0.028 ± 0.000^a^	0.029 ± 0.000^a^	0.027 ± 0.001^a^
	LSD 0.05 = 0.003				
	*T*-test	^**—**^	1.612^NS^	−1.282^NS^	0.542^NS^

FER %	Mean ± SE	3.166 ± 0.149^a^	2.816 ± 0.087^a^	2.933 ± 0.084^a^	2.766 ± 0.108^a^
	LSD 0.05 = 0.274				
	*T*-test	^**—**^	1.612^NS^	−1.282^NS^	0.542^NS^

Data are represented as mean ± SE. *T*-test values. ANOVA analysis: within each row, means with different superscript (a, b, c, or d) are significantly different at *P* < 0.05, whereas means superscripts with the same letters mean that there is no significant difference at *P* < 0.05. LSD: least significant difference; N.S: nonsignificant.

**Table 3:** Effect of administration of 20% (w/w) parsley and carob methanol extracts for 8 weeks on serum lipids in hypercholesterolemic male rats.

Parameters	Statistics	G1 negative control	G2 positive control	G3 20% w/w parsley methanol extract	G4 20% w/w carob methanol extract
STC mg%	Mean ± SE	163.50 ± 2.81^d^	273.166 ± 5.12^a^	204.33 ± 3.13^c^	234.17 ± 4.94^b^
	LSD 0.05 = 13.878				
	*T*-test	^—^	−16.06^*∗∗∗*^	9.70^*∗∗∗*^	4.60^*∗∗∗*^

STG mg/dl	Mean ± SE	134.00 ± 4.63^b^	221.17 ± 3.11^a^	178.50 ± 2.75^a^	197.50 ± 2.23^a^
	LSD 0.05 = 32.387				
	*T*-test	^—^	−13.63^*∗∗∗*^	8.993^*∗∗∗*^	7.10^*∗∗∗*^

SHDLc mg/dl	Mean ± SE	46.33 ± 0.66^a^	33.66 ± 1.49^c^	40.33 ± 0.49^b^	37.00 ± 0.57^d^
	LSD 0.05 = 3.050				
	*T*-test	^—^	6.35^*∗∗∗*^	−3.62^*∗∗*^	−1.76^NS^

SLDLc mg/dl	Mean ± SE	90.36 ± 2.62^d^	196.33 ± 5.21^a^	127.83 ± 2.57^c^	157.17 ± 4.80^b^
	LSD 0.05 = 13.194				
	*T*-test	^—^	−15.72^*∗∗∗*^	10.36^*∗∗∗*^	4.58^*∗∗∗*^

VLDLc mg/dl	Mean ± SE	26.80 ± 0.92^d^	44.23 ± 0.62^a^	35.70 ± 0.55^c^	39.50 ± 0.44^b^
	LSD 0.05 = 2.222				
	*T*-test	^—^	−13.63^*∗∗∗*^	8.99^*∗∗∗*^	7.10^*∗∗∗*^

Data are represented as mean ± SE. *T*-test values; ^*∗∗*^significant at *P* < 0.01; ^*∗∗∗*^significant at *P* < 0.001. ANOVA analysis: within each row, means with different superscript (a, b, c, or d) are significantly different at *P* < 0.05, whereas means superscripts with the same letters mean that there is no significant difference at *P* < 0.05. LSD: least significant difference; NS: nonsignificant.

**Table 4:** Effect of administration of 20% (w/w) parsley and carob methanol extracts for 8 weeks on serum liver enzymes, lactate dehydrogenase, and creatine kinase-MB in hypercholesterolemic male rats.

Parameters	Statistics	G1 negative control	G2 positive control	G3 20% w/w parsley methanol extract	G4 20% w/w carob methanol extract
ALT U/l	Mean ± SE	24.00 ± 1.06^d^	65.00 ± 0.57^a^	47.66 ± 0.55^c^	56.16 ± 0.70^b^
	LSD 0.05 = 1.471				
	*T*-test	—	−60.01^*∗∗∗*^	28.20^*∗∗∗*^	53.00^*∗∗∗*^

AST U/l	Mean ± SE	25.33 ± 1.76^d^	77.50 ± 0.99^a^	53.16 ± 0.87^c^	66.83 ± 0.70^b^
	LSD 0.05 = 3.298				
	*T*-test	—	−27.64^*∗∗∗*^	19.37^*∗∗∗*^	12.64^*∗∗∗*^

ALP U/l	Mean ± SE	160.33 ± 4.09^d^	282.00 ± 4.47^a^	188.67 ± 2.74^c^	229.50 ± 3.87^b^
	LSD 0.05 = 12.378				
	*T*-test	—	−16.83^*∗∗∗*^	18.35^*∗∗∗*^	7.39^*∗∗∗*^

Lactate dehydrogenase U/l	Mean ± SE	179.00 ± 4.50^d^	449.33 ± 3.12^a^	223.83 ± 1.70^c^	367.17 ± 12.05^b^
	LSD 0.05 = 17.474				
	*T*-test	—	−54.23^*∗∗∗*^	60.73^*∗∗∗*^	8.03^*∗∗∗*^

Creatine kinase-MB ug/ml	Mean ± SE	20.76 ± 0.81^a^	21.01 ± 0.72^a^	20.95 ± 0.78^a^	20.93 ± 0.77^a^
	LSD 0.05 = 1.970				
	*T*-test	—	−0.32^NS^	0.06^NS^	0.11^NS^

Data are represented as mean ± SE. *T*-test values; ^*∗∗∗*^significant at *P* < 0.001. ANOVA analysis: within each row, means with different superscript (a, b, c, or d) are significantly different at *P* < 0.05, whereas means superscripts with the same letters mean that there is no significant difference at *P* < 0.05. LSD: least significant difference; NS: nonsignificant.
